# Patterns of lung cancer mortality in 23 countries: Application of the Age-Period-Cohort model

**DOI:** 10.1186/1471-2458-5-22

**Published:** 2005-03-05

**Authors:** Yung-Po Liaw, Yi-Chia Huang, Guang-Wen Lien

**Affiliations:** 1 Department of Public Health, Chung Shan Medical University, Taichung, 402, Taiwan; 2 School of Nutrition, Chung Shan Medical University, Taichung, 402, Taiwan

## Abstract

**Background:**

Smoking habits do not seem to be the main explanation of the epidemiological characteristics of female lung cancer mortality in Asian countries. However, Asian countries are often excluded from studies of geographical differences in trends for lung cancer mortality. We thus examined lung cancer trends from 1971 to 1995 among men and women for 23 countries, including four in Asia.

**Methods:**

International and national data were used to analyze lung cancer mortality from 1971 to 1995 in both sexes. Age-standardized mortality rates (ASMR) were analyzed in five consecutive five-year periods and for each five-year age group in the age range 30 to 79. The age-period-cohort (APC) model was used to estimate the period effect (adjusted for age and cohort effects) for mortality from lung cancer.

**Results:**

The sex ratio of the ASMR for lung cancer was lower in Asian countries, while the sex ratio of smoking prevalence was higher in Asian countries. The mean values of the sex ratio of the ASMR from lung cancer in Taiwan, Hong Kong, Singapore, and Japan for the five 5-year period were 2.10, 2.39, 3.07, and 3.55, respectively. These values not only remained quite constant over each five-year period, but were also lower than seen in the western countries. The period effect, for lung cancer mortality as derived for the 23 countries from the APC model, could be classified into seven patterns.

**Conclusion:**

Period effects for both men and women in 23 countries, as derived using the APC model, could be classified into seven patterns. Four Asian countries have a relatively low sex ratio in lung cancer mortality and a relatively high sex ratio in smoking prevalence. Factors other than smoking might be important, especially for women in Asian countries.

## Background

Worldwide, over one million people die of lung cancer each year [1]. In the US, lung cancer is the most common cause of cancer deaths in both sexes [2], and mortality rates in women have risen 500% since 1950 [3]. In the European Union countries, although age-standardized mortality rates have decreased for most cancer sites, lung cancer mortality rates have significantly risen in women [4]. A rising death rate from lung cancer has also been observed in Taiwan. Between 1971 and 2001, age-standardized lung cancer mortality rates per 100 000 per year in Taiwan have increased sharply, from 12.66 to 32.93 among men and from 7.83 to 14.94 among women [5]. Today, in Taiwan, lung cancer is the leading cause of cancer death in women and the second leading cause in men [5].

Epidemiological studies have shown that cigarette smoking is the major cause of lung cancer in both sexes [6-8]. However, smoking habits do not seem to be the main explanation of the epidemiological characteristics of female lung cancer mortality in Asian countries [9-13], where the prevalence of smoking is relatively low but lung cancer mortality rates are relatively high. Factors other than smoking habits might contribute to the variability in lung cancer mortality.

Long-term geographical trends in cancer mortality can provide useful information to assist etiological research. However, Asian countries are often excluded from studies of geographical differences in trends in lung cancer mortality. In order to clarify the changing patterns of lung cancer mortality worldwide, we examined lung cancer trends from 1971 to 1995 among men and women for 23 countries including four from Asia – Taiwan, Japan, Singapore, and Hong Kong. In addition, we plotted the pattern of mortality rate in these countries by using the age-period-cohort (APC) model.

## Methods

We used data from the World Health Organization (WHO) and Taiwan to analyze secular trends from 1971–1995 in lung cancer mortality in both men and women. Mortality data provided by WHO were relatively incomplete in some countries, so we analyzed data from 22 countries and Taiwan. The twenty-two countries – Hong Kong, Singapore, Japan, Portugal, Poland, Italy, Cuba, Spain, Hungary, France, Greece, Finland, United States, England and Wales, Netherlands, Belgium, Canada, Australia, New Zealand, Denmark, Norway and Sweden – are members of WHO. The data for Taiwan came directly from the Office of Statistics, Department of Health in Taiwan. Since rates for the under 30-year age group are often based on few deaths, and rates for the over 80-year group might be affected by competitive death effects, only rates for the age range 30 to 79 were considered, so as to ensure adequate reliability of the estimates. Lung cancer mortality rates between 1971 and 1995 were analyzed in five consecutive five year periods (1971–1975, 1976–1980, 1981–1985, 1986–1990, and 1991–1995) and in five year age groups.

## Statistical methods

Age-standardized mortality rates (ASMR) were calculated using the world population for 1976 as the reference [14]. Percent changes in the ASMR were calculated as [(ASMR_1991–1995 _- ASMR_1971–1975_) / (ASMR_1971–1975_)] × 100.

In order to apply the APC model, the matrix of age-specific death rates was calculated for each 5-year calendar period (from 1971–1975 to 1991–1995) and age group (from 30–34 to 75–79). The effect of period of death in the APC model was evaluated by a log-linear Poisson model with a modified method as described by Osmond and Gardner [15]. Briefly, the estimate of period effect results from minimizing the weighted sum of the Euclidean distances from the three possible two-factor models (age/period; age/cohort; period/cohort). The weights used in the minimization process were based on the goodness-of-fit measures of each two-factor model. In this study, these were taken as the inverse of the deviance statistics. The sum of period effects were constrained to be zero. These "effects" can be interpreted as logarithms of "relative" risks. These relative risks were estimated separately for men and women. A computer program written in the SAS/IML language [16] was developed to perform the above calculations.

## Results

Age-standardized mortality rates from lung cancer per 100 000 population per year in 23 countries for 1971 to 1995 are listed for men in Table 1 and for women in Table 2. Trends in the ASMR varied by sex. From 1971 to 1995, in men, the rates progressively increased in nine countries (Portugal, Hungary, Taiwan, Spain, Poland, Japan, Norway, France and Greece), progressively decreased in two countries (England and Wales and Finland) and increased then declined in the others. In women, rates increased between 1971–1975 and 1991–1995 in 23 countries except for Hong Kong, Cuba, and Spain, with the highest increasing rate observed in the Netherlands (223.46%).

**Table 1:** Age-standardized mortality rate (per100 000 person years) from lung cancer in males in 23 countries, 1971–1995

	**ASMR**						
	
**Country**	1971–1975	1976–1980	1981–1985	1986–1990	1991–1995	Percent increase*	Rank^$^
Portugal	31.88	40.51	45.96	54.40	59.48	86.57	1
Hungary	97.10	116.05	138.49	161.34	180.91	86.31	2
Taiwan	32.08	39.80	49.95	54.24	58.44	82.17	3
Spain	56.92	68.84	80.68	93.80	101.49	78.30	4
Poland	93.91	113.65	135.11	152.23	158.34	68.60	5
Japan	38.88	46.55	53.40	56.96	59.44	52.87	6
Norway	45.20	53.24	62.61	65.43	67.36	49.03	7
France	74.81	87.45	93.16	99.32	100.08	33.77	8
Greece	79.55	93.63	99.23	103.77	105.44	32.54	9
Italy	94.59	110.47	123.02	125.63	117.10	23.80	10
Hong Kong	94.96	118.63	116.24	116.89	110.13	15.97	11
Canada	96.68	108.38	116.23	118.13	107.71	11.42	12
USA	109.60	117.87	121.16	120.33	115.23	5.13	13
Denmark	101.45	109.36	116.83	113.21	103.74	2.26	14
Sweden	48.34	52.24	50.10	48.56	48.21	-0.27	15
Singapore	92.34	114.24	115.29	102.90	91.47	-0.95	16
Belgium	146.33	163.41	166.59	155.70	144.87	-1.00	17
Cuba	75.86	76.35	76.59	76.16	72.90	-3.90	18
Netherlands	151.10	162.14	160.49	148.73	129.40	-14.36	19
Australia	99.14	100.74	100.11	90.81	80.86	-18.44	20
New Zealand	99.81	104.55	100.52	93.07	79.31	-20.54	21
England and Wales	159.59	152.45	136.82	120.40	101.21	-36.58	22
Finland	142.74	142.51	126.97	105.58	90.20	-36.81	23

*percent increase (%) = 100 × (ASMR_1991–1995 _- ASMR_1971–1975_) / (ASMR_1971–1975_)

^$ ^Rank by percent increase

**Table 2:** Age-standardized mortality rate (per100 000 person years) from lung cancer in females in 23 countries, 1971–1995

	**ASMR**						
	
**Country**	1971–1975	1976–1980	1981–1985	1986–1990	1991–1995	percent increase*	Rank^$^
Netherlands	8.42	11.06	15.46	20.80	27.24	223.46	1
Norway	8.86	11.02	15.61	21.21	26.42	198.24	2
Denmark	21.25	29.28	40.86	49.78	58.41	174.91	3
Canada	17.94	25.44	34.93	43.53	49.13	173.81	4
USA	24.96	33.85	43.41	51.50	56.51	126.46	5
Hungary	16.75	19.34	22.99	29.11	36.83	119.89	6
Sweden	11.36	13.47	16.96	20.68	24.50	115.72	7
Poland	11.40	13.66	16.41	19.68	22.90	100.83	8
Australia	14.91	19.70	23.61	26.47	28.47	90.99	9
Belgium	10.74	12.31	13.79	16.45	19.86	84.90	10
New Zealand	22.58	26.88	30.83	36.53	38.00	68.29	11
France	7.07	7.44	8.42	9.97	11.89	68.19	12
Finland	8.71	11.35	12.39	13.48	14.54	67.02	13
Taiwan	16.03	19.55	23.13	25.69	26.43	64.94	14
Italy	10.69	12.12	13.51	15.09	16.23	51.86	15
England and Wales	30.45	35.75	40.06	43.52	42.84	40.68	16
Portugal	6.74	7.14	7.97	8.56	9.43	39.97	17
Japan	11.90	13.60	14.97	15.35	15.73	32.25	18
Greece	12.64	13.57	13.21	13.86	14.28	12.97	19
Singapore	29.66	34.34	37.07	36.01	31.25	5.36	20
Hong Kong	44.18	48.99	48.01	48.32	43.14	-2.34	21
Cuba	27.87	26.63	26.90	27.77	27.03	-3.02	22
Spain	8.54	7.99	7.32	7.01	7.58	-11.27	23

* percent increase(%) = 100 × (ASMR_1991–1995 _- ASMR_1971–1975_) / (ASMR_1971–1975_)

^$ ^Rank by percent increase

Table 3 shows the sex ratio (male:female) of the ASMR for lung cancer for five consecutive five- year periods in 23 countries. The sex ratio was greater than one in each five-year period, indicating that the ASMR from lung cancer was higher in men than in women. Among the 23 countries, the trend in the sex ratio gradually decreased for the whole period in most countries except for Spain, France, Italy, Poland, Greece, Portugal, Hungary, Cuba and the Asian countries. For example, in 1971–1975, the highest sex mortality ratio was seen in the Netherlands with a sex ratio of 17.95, the ratio then gradually decreasing to a value of 4.75 by 1991–1995. The change in the sex ratio of mortality in the Netherlands might be due to the increase and then decrease in male lung cancer mortality and simultaneously to the increase in female lung cancer mortality. On the other hand, the ratio gradually increased in Spain. This might be due to Spain having the the fourth highest increase in male lung cancer mortality accompanied by a progressive decrease in female lung cancer mortality from 1971–1975 through 1986–1990, followed by a slight increase.

**Table 3:** Sex ratio of the age-standardized mortality rate from lung cancer in 23 countries, 1971–1995

	**Ratio**							
		
**Country**	1971–1975	1976–1980	1981–1985	1986–1990	1991–1995	Mean	Range	Rank*
Belgium	13.62	13.27	12.08	9.46	7.29	11.15	6.33	1
Netherlands	17.95	14.67	10.38	7.15	4.75	10.98	13.20	2
Finland	16.40	12.56	10.25	7.83	6.20	10.65	10.20	3
Spain	6.66	8.62	11.02	13.39	13.39	10.62	6.73	4
France	10.58	11.76	11.06	9.97	8.42	10.36	3.34	5
Italy	8.85	9.12	9.11	8.33	7.21	8.52	1.91	6
Poland	8.24	8.32	8.23	7.73	6.91	7.89	1.41	7
Greece	6.30	6.90	7.51	7.49	7.39	7.12	1.21	8
Portugal	4.73	5.67	5.77	6.36	6.31	5.77	1.63	9
Hungary	5.80	6.00	6.03	5.54	4.91	5.66	1.12	10
Australia	6.65	5.11	4.24	3.43	2.84	4.45	3.81	11
Norway	5.10	4.83	4.01	3.09	2.55	3.92	2.55	12
England and Wales	5.24	4.26	3.42	2.77	2.36	3.61	2.88	13
Canada	5.39	4.26	3.33	2.71	2.19	3.58	3.20	14
Japan	3.27	3.42	3.57	3.71	3.78	3.55	0.51	15
New Zealand	4.42	3.89	3.26	2.55	2.09	3.24	2.33	16
Denmark	4.77	3.74	2.86	2.27	1.78	3.08	2.99	17
Sweden	4.26	3.88	2.95	2.35	1.97	3.08	2.29	18
Singapore	3.11	3.33	3.11	2.86	2.93	3.07	0.47	19
USA	4.39	3.48	2.79	2.34	2.04	3.01	2.35	20
Cuba	2.72	2.87	2.85	2.74	2.70	2.78	0.17	21
Hong Kong	2.15	2.42	2.42	2.42	2.55	2.39	0.40	22
Taiwan	2.00	2.04	2.16	2.11	2.21	2.10	0.21	23

*Rank by mean

The mean values of the sex ratio of the ASMR from lung cancer in Taiwan, Hong Kong, Cuba, Singapore, and Japan were 2.10, 2.39, 2.78, 3.07, and 3.55, respectively, with a range of 0.17 to 0.51 over the five-year periods. These values were not only relatively constant over time, but were also lower than seen in the western countries. For example, the lowest sex ratio of 2.10 was seen in Taiwan, with the sex ratio remaining a relatively constant value over the entire period.

**Figure 1:**
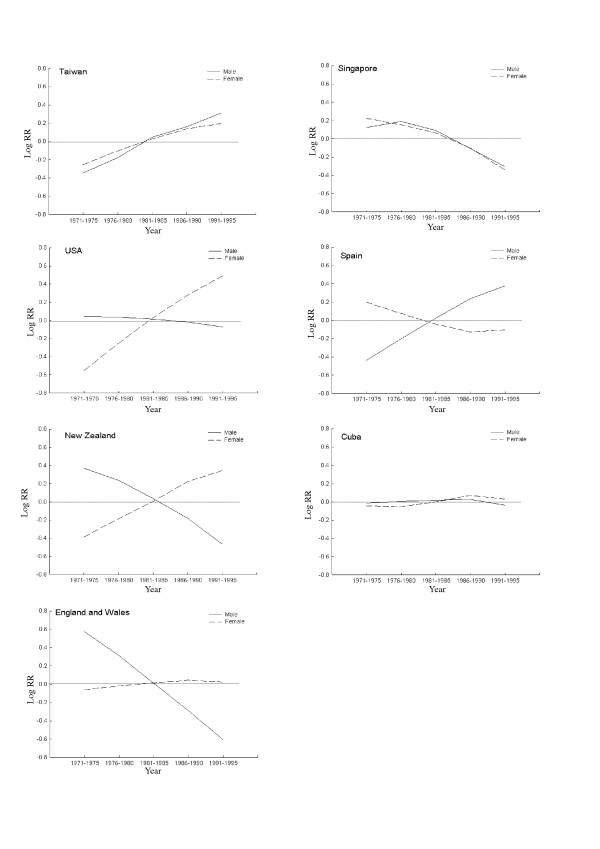
Secular trend in the relative risk (RR) of dying from male and female lung cancer, 1971–1995, based on analyses using the age-period-cohort model in Taiwan, England and Wales, New Zealand, USA, Singapore, Spain and Cuba

Only age is adjusted when the ASMR is calculated. However, both age and cohort effect are adjusted in the APC model. The period effects for males and females from the APC model applied to the data from the 23 countries could be classified into seven patterns: 1) an increasing trend in both sexes, seen in Taiwan (Figure 1), Norway, Japan, Hungary, and Portugal; 2) a sharply increasing trend in women, with little change seen in men seen in USA (Figure 1), Sweden, Poland, Italy, Canada, Belgium, Denmark and France; 3) a sharply increasing trend in women, and a sharply decreasing trend in men, seen in New Zealand (Figure 1), Finland, Australia and Netherlands; 4) a more gradual increasing trend in women, but a sharply declining trend in men, seen only in England and Wales (Figure 1); 5) a decreasing trend in both sexes, seen in Singapore (Figure 1) and Hong Kong; 6) a decreasing and then a gradually increasing trend in women, but a sharply increasing trend in men, seen in Spain (Figure 1) and Greece; and 7) a relatively steady trend in both sexes, seen only in Cuba (Figure 1). In most countries, the long-term trend in the period effect as derived from the APC model was similar to the trend of ASMR. It is worth noting that the trend in the ASMR for female lung cancer increased and then declined in Singapore and Hong Kong. After adjusting for the cohort effect, however, a decreasing trend in the period effect was observed in Singapore and Hong Kong.

## Discussion

We found that the sex ratio in lung cancer mortality varied over time and geographically. After adjusting for age and cohort effects, seven patterns could be identified using the APC model, indicating that some countries had a similar trend in lung cancer mortality.

Koo and Ho [17] indicated that smoking was a strong risk factor in the west and worldwide where there were high rates of smoking in men. We appreciate that lung cancer mortality rates for a given year depend on smoking habits over a period before that year; however, it is not possible to get data on smoking prevalence before 1975 from WHO. Therefore, based on the smoking prevalence obtained from World Health Organization (Table 4), the first, second, third, and fifth highest sex ratios (male:female) of smoking prevalence among the 23 countries were in Taiwan, Hong Kong, Singapore, and Japan, respectively. However, the sex ratios of ASMR from lung cancer in the four Asian countries were significantly lower than in the western countries. That is, the four Asian countries have a relatively low sex ratio in lung cancer mortality and a relatively high sex ratio in smoking prevalence. This fact is of particular interest.

**Table 4:** Smoking prevalence in males and females, and their sex ratio, in 23 countries

**Country**	**Prevalence**				
	
	Male	Female	Male: female ratio	Rank*	Data source^$^
Taiwan	55.1	3.3	16.7	1	Adult (18 years & older), 1996
Hong Kong	27.1	2.9	9.3	2	Adult (15 years & older), 1998
Singapore	26.9	3.1	8.7	3	Adult (18–64 year olds), 1998
Portugal	30.2	7.1	4.3	4	Adult (15 years & older), 1995–1996
Japan	52.8	13.4	3.9	5	Adult (15 years & older), 1998
Poland	39.0	19.0	2.1	6	Adult, 1998
Italy	32.2	17.3	1.9	7	Adult (14 years & older), 1998
Cuba	48.0	26.3	1.8	8	Adult (15 years & older), 1995
Spain	42.1	24.7	1.7	9	Adult (16 years & older), 1997
Greece	46.0	28.0	1.6	10	Adult, 1994–1998
Hungary	44.0	27.0	1.6	11	Adult (18 years & older), 1998–1999
France	39.0	27.0	1.4	12	Adult (18 years & older), 1997
Finland	27.0	20.0	1.4	13	Adult (15–64 year olds), 1999
USA	27.6	22.1	1.3	14	Adult (18 years & older), 1997
Netherlands	37.0	30.0	1.2	15	Adult (15 years & older), 1998
Belgium	31.0	26.0	1.2	16	Adult (15 years & older), 1999
Canada	27.0	23.0	1.2	17	Adult (15 years & older), 1999
Australia	27.1	23.2	1.2	18	Adult (16 years & older), 1995
New Zealand	26.0	24.0	1.1	19	Adult (15 years & older), 1998
Denmark	32.0	30.0	1.1	20	Adult (14 years & older), 1998
England and Wales	29.0	28.0	1.0	21	Adult (16 years & older), 1996
Norway	33.7	32.3	1.0	22	Adult (16–74 year olds), 1998
Sweden	17.1	22.3	0.8	23	Adult (16–84 year olds), 1998

*Rank by Male: female ratio

^$^Data were obtained from the World Health Organization

Dietary fat consumption has been found to be positively related to lung cancer mortality [18-20]. Our data from Japan, Taiwan and Cuba women (on Tables 2 and 5) also indicated that the percent increase of fat consumption was positively related to the percent increase of ASMR by using the Spearman's rank correlation coefficient. Further study of factors other than smoking, like fat intake, on lung cancer mortality seems warranted, especially for women in Asian countries (Japan and Taiwan).

**Table 5:** Changes in annual per caput fat consumption in 23 countries

	Fat Consumption*			
	
**Country**	1970	1990	Percent increase	Rank^&^
Taiwan	38.0	136.8	260.0%	1
Hong Kong	71.4^$^	135.6^#^	89.9%	2
Spain	88.9	137.0	54.1%	3
Portugal	78.6	120.6	53.4%	4
Japan	54.6	79.3	45.2%	5
Greece	101.9	138.4	35.8%	6
Hungary	115.3	153.5	33.1%	7
Italy	114.4	151.0	32.0%	8
France	126.4	161.3	27.7%	9
Cuba	67.6	85.1	25.9%	10
New Zealand	115.4	134.7	16.7%	11
USA	119.6	138.8	16.1%	12
Canada	113.7	127.1	11.8%	13
Australia	117.8	130.6	10.9%	14
Netherlands	132.0	140.9	6.7%	15
Poland	103.9	110.3	6.2%	16
Sweden	116.8	122.6	5.0%	17
Finland	123.6	124.2	0.5%	18
Norway	131.6	127.7	-3.0%	19
England & Wales	141.7	135.8	-4.2%	20
Denmark	140.7	132.6	-5.8%	21
Belgium	-	-	-	-
Singapore	-	-	-	-

*Data were obtained from FAO

^&^Rank by percent increase

^$^:1961,^#^: 1995

## Conclusion

Period effects for both men and women in 23 countries, as derived using the APC model, could be classified into seven patterns. The four Asian countries have a relatively low sex ratio in lung cancer mortality and a relatively high sex ratio in smoking prevalence. Factors other than smoking might be important, especially for women in Asian countries.

## Competing interests

The author(s) declare that they have no competing interests.

## Authors' contributions

YPL was responsible for the development of intellectual content and the study design, collected and analyzed the data, interpretation of the results, manuscript drafting and the critical revisions of manuscript. YCH was responsible for the development of intellectual content, interpretation of the results and manuscript drafting. GWL was responsible for data coding and entry and statistical analyses.

## Pre-publication history

The pre-publication history for this paper can be accessed here:


